# Supplementary material for: Conditional chemoconnectomics (cCCTomics) as a strategy for efficient and conditional targeting of chemical transmission
Source: eLife. 2024 Apr 30;12:RP91927. doi: 10.7554/eLife.91927 (PMC11060718; doi:10.7554/eLife.91927)
Supplement: Figure 3—source data 3. [file elife-91927-fig3-data3.zip › Figure 3 source data3 part 2.zip!/Figure 3-Source data 3-part2/1002-9-CRISPResso_Report_e53dd8m5.zip!/CRISPResso_on_1002_12/2b.Nucleotide_percentage_quilt_around_sgRNA_gtatctgtccagctacgtga.pdf]

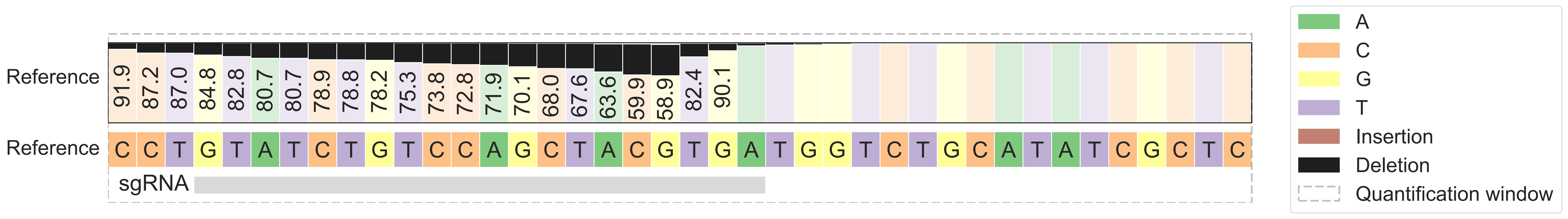

Reference
91.9
87.2
87.0
84.8
82.8
80.7
80.7
78.9
78.8
78.2
75.3
73.8
72.8
71.9
70.1
68.0
67.6
63.6
59.9
58.9
82.4
90.1
Reference
C C T G T A T C T G T C C A G C T A C G T G A T G G T C T G C A T A T C G C T C
sgRNA
A
C
G
T
Insertion
Deletion
Quantification window
